# The Effects of Total Flavonoids from Buckwheat Flowers and Leaves on Renal Damage and PTP1B Expression in Type 2 Diabetic Rats

**Published:** 2011

**Authors:** Jin-Xiu Chu, Zhi-Lu Wang, Shu-Ying Han

**Affiliations:** a *Department of Pharmacology, North China Coal Medical University, Tangshan, China.*; b *Office of Academic Affairs, Tangshan Teacher’s College, Tangshan, China.*

**Keywords:** Buckwheat, Flavonoids, Type 2 diabetes mellitus, Renal damage, PTP1B

## Abstract

Clinical data showed consumption of buckwheat played a very positive role in the relief of diabetes and its complications. The purpose of this study was to explore the effects and mechanisms of the overall flavonoids from buckwheat flowers and leaves (TFBFL) on renal damage in type 2 diabetes mellitus (T2DM) rats. Seventy male Wistar rats were selected. Ten rats were randomly allocated into a normal group and the other sixty were intragastrically injected with a lipid emulsion and small doses of alloxan to induce the T2DM model. T2DM inducement was judged by the fasting blood glucose (FBG) and oral glucose tolerance test (OGTT). Those whose FBG was ≥ 16.7 mmol/L and less tolerant to glucose were considered as being T2DM rats. These rats were then randomly divided into a groups termed: model (purified water, 5 mL.kg^-1 ^. d^-1^), BNPL (positive control) (Benazepril, 4 mg.kg^-1 ^. d^-1^), L-TFBFL (TFBFL 100 mg.kg^-1 ^. d^-1^), M-TFBFL (TBFL 200 mg.kg^-1 ^. d^-1^) and H-TFBFL (TFBFL 400 mg.kg^-1 ^. d^-1^). Each group then received medication for a period of 4 weeks. The normal rats were treated with purified water in a synchronous manner. Subsequently, FBG, plasma insulin (INS), OGTT, 24 h urinary protein output, blood and urinary creatinine content were assayed. Then the insulin sensitive index (ISI), bilateral kidney index, and creatinine clearance rate (Ccr) were calculated. Renal morphological changes and expression of protein tyrosine phosphatase 1B (PTP1B) in the kidneys were observed. TFBFL lowered FBG, improved insulin resistance, caused Ccr, and renal morphological changes, down-regulated the expression of PTP1B in T2DM rats and showed dose-dependence. TFBFL had a significant protective effect on renal damage in T2DM rats. This effect may be due to lowering blood glucose and diminishing renal damage by inhibiting PTP1B expression.

## Introduction

Diabetes Mellitus is a common metabolic disease of the endocrine system with more than 95% of the patients having Type 2 Diabetes Mellitus (T2DM). Diabetic nephropathy is one of its severe complications and the main cause of chronic renal dysfunction. The incidences of patients with terminal stage renal function failure and subsequent diabetic nephropathy have been increasing yearly. The pathogenic mechanism causing the disease remains unclear and there is a lack of effective treatment methods. As such it is very important to discover the etiological factors of diabetic nephropathy. Also methods of prevention and treatment should be found to actively improve the quality of life of diabetic.

patients in the process extending their life. Chinese herbal medicine and natural compounds effective on Diabetes Mellitus and diabetic nephropathy have been in the news recently. Total flavonoids from buckwheat flowers and leaves (TFBFL) are active ingredients extracted from *Fagopyrum esculentum *Moench, also called sweet buckwheat. Studies found TFBFL reduced blood glucose, lowered blood lipid, inhibited the formation of products from the glycation of proteins *in-vitro *and *in-vivo *and so on (1, 2). Based on these findings, this study further observed the effect of TFBFL on renal damage and PTP1B expression in T2DM rats exploring its possible mechanisms of action.

## Experimental

*Preparation of TFBFL*

The preparation of TFBFL was outlined in the literature (3). Briefly, buckwheat flowers and leaves were collected in late autumn from Ku Lun, Inner Mongolia (China). The air-dried flowers and leaves were ground, refluxed for 30 min with water (three times) then dried in an oven at 70˚C to obtain a powder extract. The water extract was dissolved in ethanol and its impurities were removed by filtration. The ethanol was recycled by rotate evaporation. The filtrate was concentrated and dried to obtain TFBFL (amount to 98%). It was confirmed as being flavonoids by the Institute of Materia Medica, Chinese Academy of Medical Sciences.

*Establishment of T2DM model*

The T2DM model was established following the literature (4, 5) with some alterations. The rats were intragastrically injected with 2 mL.kg^-1^.d^-1^ lipid emulsion (lard 2 g, cholesterol 0.5 g, sodium glutamate 0.1 g, sucrose 0.5 g, fructose 0.5 g, Tween 2 mL, glycol propylene 3 mL, propylthiouracil 0.1 g, distilled water added to achieve 100 mL) daily. From the 14^th^ day onwards, small doses of alloxan was added by intraperitoneal injection once every other day thrice (120 mg· kg^-1^ for the first time, 100 mg·kg^-1^ for the other two), and 0.4 U of insulin (Wanbang Biopharma, China) was intraperitoneally injected 15 min later. 25% glucose solution (10 mL·kg^-1^) was then administrated intragastrically 2.5 h and 5 h later respectively. Seventy-two hours following the last injection of alloxan, the fasting blood glucose (FBG) level was with taken using a blood glucose monitor (Abbott Laboratories, USA) and oral glucose tolerance test (OGTT). Those with a FBG of ≥16.7 mmol/L and found to be less tolerant to glucose were considered as being type 2 diabetic rats. This study was in accordance with the Principles of Laboratory Animal Care.

*Experimental groups and medication*

Seventy healthy male Wistar rats (provided by Institute of experimental animals, Beijing Med Healthcare, Inc,China. Certificate No. SCXKII-00-0006.) weighing 200 ± 20 g were selected. Ten of them were randomly allocated into a normal group and 56 of the other sixty were successfully T2DM induced. The model formated rats were randomly divided into 5 groups. The groups and medication administered were as follows: Normal group: 10 rats, intragastrically injected with purified water, 5 mL.kg^-1^.d^-1^ for 4 w. Model group: 12 rats, intragastrically injected with purified water, 5 mL.kg^-1^.d^-1^ for 4 w. BNPL (Positive control) group: 11 rats, intragastrically injected with BNPL (Beijing Novartis Pharma Ltd, Batch number: X0256), 4 mg.kg^-1^.d^-1^ for 4 w. L-TFBFL group: 11 rats, intragastrically injected with TFBFL, 100 mg.kg^-1^.d^-1^ for 4 w. M-TFBFL group: 11 rats, intragastrically injected with TFBFL, 200 mg.kg^-1^.d^-1^ for 4 w. H-TFBFL group: 11 rats, intragastrically injected with TFBFL, 400 mg.kg^-1^ . d^-1^ for 4 w.

It was found in the initial research (6) that the total flavones of the *buckwheat *flower at the doses of 100 mg.kg^-1^ . d^-1^, 200 mg·kg^-1^ . d^-1^ and 400 mg.kg^-1^.d^-1^ inhibited the formation of AGEs of proteins both *in-vivo *and *in-vitro*. The concentrations of TFBFL were set with reference to literatures and trial experiments.

*OGTT, FBG and INS assay*

All the rats were fasted for 12 h following the final administration and the FBG in caudal vein was measured with a blood glucose monitor. Then, 20% glucose solution was intragastrically injected at 10 mL.kg^-1^. Subsequently, the blood glucose was measured at 30 min, 60 min and 120 min respectively. Following which, blood was collected from abdominal aorta and the plasma INS was detected by radioimmunoassay (Atom-hitechnology HTA Co., Ltd). The ISI was calculated using the formula: ISI = Ln [1/ (INS×FBG)] (7)

*Observation of renal damages*

Renal damage was evaluated by determining the urinary protein output, blood and urinary creatinine, calculating Ccr and kidney indexes, and by observing renal morphological changes with the naked eye and using light microscopy (8, 9). Following the 4 weeks of medications, urine was gathered for 24 h using metabolism cages. The content of urinary protein was tested using the Bradford method (10). Blood and urinary creatinine were measured with the aid of the Hitachi 7150 Fully Automated Biochemical Assay Instrument (Japan). The ccr was calculated with the following the formula: Ccr = [concentration of urinary creatinine (mg/mL) × 1 min urinary volume (mL)]/ concentration of blood creatinine (mg/mL). The kidney index (mg/g) was the ratio of the bilateral kidneys weight (mg) to body weight (g). Part of the kidney was fixed with 10% formalin, embedded in paraffin, cut into 5 μm slices and stained with H.E. to observe any pathological changes.

*Immunohistochemistry (IHC) method for expression of PTP1B*

The expression of protein tyrosine phosphatase 1B (PTP1B) was measured by means of the immunohistochemistry method according to the kit instructions (Advanced Technology & Industrial Co., Ltd. China). The paraffin sections were dealt with as follows: deparaffinization → rinsing with buffer → target retrieval → blocking endogenous peroxidase with 3 %H_2_O_2_ for 10 min → rinsing with Buffer → adding antibody-I (rabbit anti-mouse) working solution overnight in 4°C wet box, for negative control adding PBS instead → rinsing with PBS for 3 times (3 min each) → adding antibody-II (goat anti-rabbit) and incubating for 10 min at 37°C → rinsing with PBS for 3 times → adding SABC and incubating for 15 min at 37°C → rinsing with PBS for 3 times → adding DAB chromogenic agent → rinsing with distilled water → adding hematoxylin for 5 min → rinsing with tap water → covering cover slips with aqueous resin. The positive cells showed up as brown. 6 visual fields were selected randomly from each slice and their optical density (OD) was analyzed using the Motic Med 6.0 digital image matching analytic systems (Beijing University of Aeronautics and Astronautics, China). The average OD values were calculated to compare the expression of PTP1B with a higher OD indicating a elevated expression of PTP1B.

*Statistical analysis*

Statistical analysis was performed with the aid of the SPSS10.0 statistical program. The data was expressed as mean ± SD and variable values were compared with the ANOVA test. p < 0.05 was considered as having a difference. p < 0.01 was considered to having a significant difference.

## Results and Discussion

In comparison with the normal group, the FBG and INS of the model group increased remarkably. Whereas the ISI significantly decreased, showing that the diabetic insulin resistance model similar to T2DM had been induced. The model was a modification from that of Ai Jing et al (4, 5) and exhibited a higher success rate (93.3%). TFBFL decreased FBG dose-dependently and was statistically different within the H-TFBFL group (p < 0.05). Though TFBFL decreased the INS without showing any statistically significance, it did nevertheless increase the ISI significantly. See Table 1.

**Table 1:** Effects of TFBFL on FBG, INS and ISI in T2DM rats (Data shown as mean ± SD).

**Group**	**n**	**Dose** **mg.kg** ^-1^ **.d** ^-1 ^	**FBG** **mmol.L** ^-1 ^	**INS** **μmol.L** ^-1^	**ISI**
Normal	10		5.18 ± 1.17	11.62 ± 1.74	-4.07 ± 0.44
Model	12		16.75 ± 3.22^b^	13.27 ± 2.35^a^	-5.43 ± 0.38^b^
BNPL	11	4	13.48 ± 3.14	12.75 ± 2.41	-5.15 ± 0.42
L- TFBFL	11	100	13.26 ± 4.02	12.78 ± 2.26	-5.15 ± 0.35
M-TFBFL	11	200	13.17 ± 3.18	13.11 ± 2.34	-5.05 ± 0.36
H- TFBFL	11	400	12.11 ± 3.02 ^c^	12.21 ± 2.11	-4.96 ± 0.33^c^

^a^p < 0.05, ^b^p < 0.01 *vs *normal group; cp < 0.05 *vs *model control

**Figure 1:**
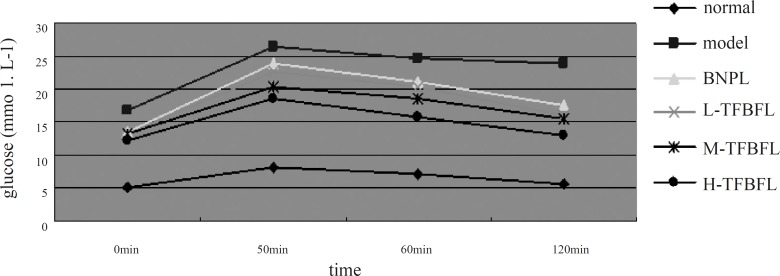
Effects of TFBFL on glucose tolerance in T2DM rats

The results illustrated in Figure 1 indicated that the normal group’s blood glucose level almost recovered 120 min following the glucose injection. The blood glucose levels of the model group at different points were higher than those of the normal group These levels remained very high at 120 min, which was in accordance with type 2 diabetic insulin resistance observed in clinics. TFBFL could improve glucose tolerance to a certain extent. The H-TFBFL group showed a significant difference compared with the model group (p < 0.01).

The results from Table 2 showed that the kidney indexes and 24 h urinary protein output in the model group were remarkably higher than those of the normal group yet the Ccr was much lower. Compared with control model, the kidney indexes and 24 h urinary protein output within the TFBFL groups decreased while the Ccr increased significantly (p < 0.01).

**Table 2:** Effects of TFBFL on kidney index , 24 h urinary protein content and Ccr in T2DM rats (Data shown as mean ± SD**).**

**Group**	**n**	**Dose** **(mg.kg** ^-1^ **.d** ^-1^ **)**	**Kidney index** **(mg.g** ^-1^ **)**	**24 h urinary protein** **(mg)**		**Ccr** **(mL.min** ^-1^ **)**
Normal	10		7.41 ± 0.73	0.36 ± 0.09	1.73 ± 0.12	
Model	12		11.73 ± 1.58^b^	1.96 ± 0.11^b^	1.01 ± 0.09^b^	
BNPL	11	4	8.87 ± 1.34^d^	1.07 ± 0.10^d^		1.42 ± 0.15^d^
L- TFBFL	11	100	10.55 ± 1.29	1.65 ± 0.17		1.08 ± 0.14
M-TFBFL	11	200	10.11 ± 1.10^c^	1.43 ± 0.15^c^		1.24 ± 0.13
H- TFBFL	11	400	9.79 ± 1.23^d^	1.34 ± 0.14^d^		1.35 ± 0.11^d^

^b^p < 0.01 *vs *normal group, ^c^p < 0.05, ^d^p < 0.01 *vs *model control

**Figure 2:**
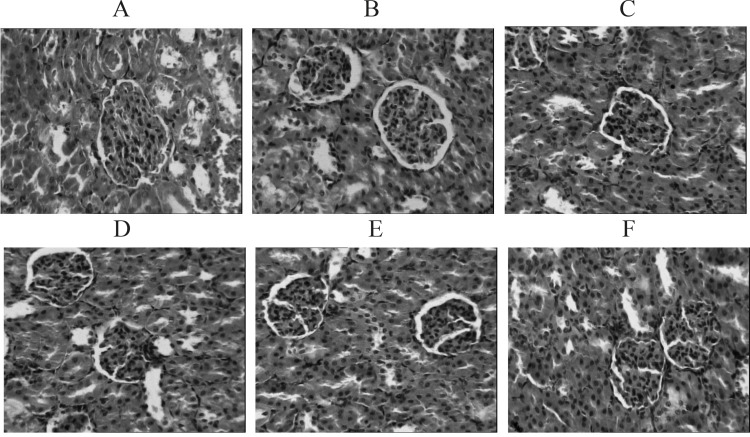
H.E. stained kidney tissue. (×100). A: Normal group. B: Model group. C: BNPL group. D: L-TFBFL group. E: M-TFBFL group. F: H-TFBFL group

In normal group, the kidneys were presented as vivid red with a smooth surface. In the model group, most of the kidneys showed up as grey white along with gathered dimensions. Microscopically the glomeruli of the kidneys showed atrophies along with an enlarged cyst cavities, accompanied by narrow lumens caused by the hyperplasia and thickening of the inner lining of renal arteries. The spotty inflammation was detected within the renal interstitial tissues. In both the TFBFL and BNPL groups, the shape and structure of the kidneys observed through microscopy showed improvement. In particular this was the case for the H-TFBFL and BNPL groups. See Figure 2 A - F.

The incidence of Diabetes Mellitus is increasing every year and has become a threatening chronic disease with a wide variety of complications. Many severe complications such as liver and renal dysfunction, cardiovascular and cerebrovascular diseases as well as nervous lesions occur in patients suffering with Diabetes Mellitus. Diabetic nephropathy is one of the complications of T2DM and is the initial trigger of end-stage renal disease in the USA and many European countries amounting to between 25% ~ 42% (11, 12). The results indicated that TFBFL could reduce blood glucose dose-dependently, improve glucose tolerance, decrease 24 h urinary protein output and renal indexes and lessen morphological manifestations of kidney tissue. Leading to the suggestion that TFBFL conveyed a protective effect on renal damage caused by T2DM. TFBFL was proven to contain 85% rutin and 3% quercetin. In all possibility rutin was responsible for the contributed effects.

**Figure 3:**
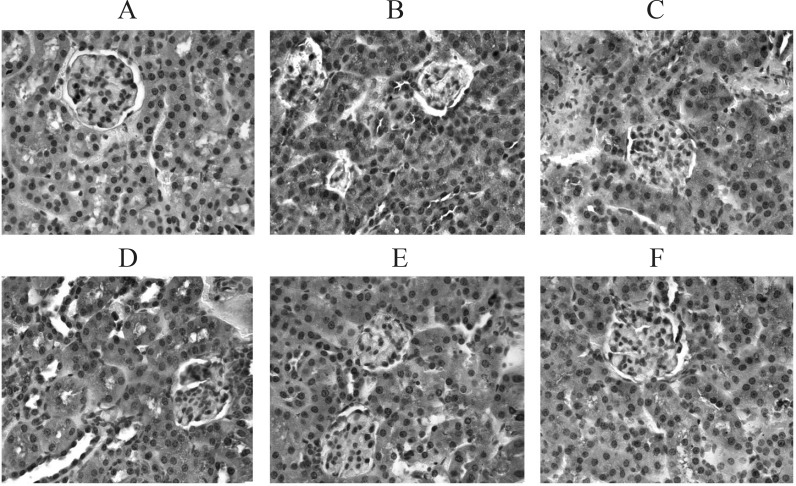
PTP1B expression in kidney tissue. (IHC, ×200). A: Normal group B: Model group. C: BNPL group. D: L-TFBFL group. E: M-TFBFL group. F: H-TFBFL group

PTP1B proved to be related to the development and progression of T2DM, and to be one of the key factors causing insulin resistance (13, 14). PTP1B is a negative regulatory factor of the insulin signal chain. Its increased activity and /or expression can down-regulate the signal transduction of insulin thus inhibiting the uptake of glucose and glycogen synthesis (15) and in turn leading to insulin resistance and a diabetic state. Literature (16) reported that the expression of PTP1B increased within the liver, skeletal muscles and fat tissue when T2DM or insulin resistance occurred. At present, there are no reports on whether or not there is excessive expression of PTP1B within the kidney tissue of T2DM rats and its influence on the development and progression of diabetic nephropathy could be ascertained. This study found that the expression of PTP1B within the renal tubules and interstitial tissues of the model group was visibly higher than that of the normal group which only showed a few positive granular particles. This would lead to glucose utilization disturbance and cause renal damage by yielding excessive final products of protein glycation in all probability. Further investigation will be undertaken. TFBFL can evidently inhibit the expression of kidney PTP1B in T2DM rats (p < 0.05, p < 0.01) (Figure 3. A – F, Table 3).The inhibitory effect of TFBFL on PTP1B expression within the renal tissue might be one of the mechanisms TFBFL employs to improve glucose tolerance and ISI leading to an improvement in renal function.

**Table 3:** Effects of TFBFL on PTP1B expression in kidneys of T2DM rats (Data shown as mean ± SD).

**Group**	**n**	**Dose (mg.kg** ^-1^ **.d** ^-1^ **)**	**OD**
Normal	6		0.16 ± 0.04
Model	6		0.25 ± 0.03^b^
BNPL	6	4	0.19 ± 0.05 ^d^
L- TFBFL	6	100	0.21 ± 0.03
M-TFBFL	6	200	0.20 ± 0.04^c^
H- TFBFL	6	400	0.19 ± 0.02^d^

^b^p < 0.01 *vs *normal group, ^c^p < 0.05, ^d^p < 0.01 *vs *model control

A great deal of experimental studies indicated that flavonoids had many pharmacological activities (17-20) and that they acted at multiple targets sites in many ways including having multiple mechanisms. Many of them are currently under investigation. Some inhibitors of PTP1B have been reported (21-23) but none of them were reported as being used in the clinical treatment of T2DM. Our results provide a theoretical and experimental basis for exploiting TFBFL as a prevention and treatment drug against Diabetes Mellitus and its complications, in addition to being a health care food supliment.
